# Heart-brain axis: Association of congenital heart abnormality and brain diseases

**DOI:** 10.3389/fcvm.2023.1071820

**Published:** 2023-03-29

**Authors:** Leihao Sha, Yajiao Li, Yunwu Zhang, Yusha Tang, Baichuan Li, Yucheng Chen, Lei Chen

**Affiliations:** ^1^ Department of Neurology, Joint Research Institution of Altitude Health, West China Hospital, Sichuan University, Chengdu, China; ^2^ Department of Cardiology, West China Hospital, Sichuan University, Chengdu, China; ^3^ Fujian Provincial Key Laboratory of Neurodegenerative Disease and Aging Research, Institute of Neuroscience, School of Medicine, Xiamen University, Xiamen, China

**Keywords:** brain disease, congenital heart abnormality, patent foramen ovale, atrial septal defect, pulmonary arteriovenous fistula

## Abstract

Brain diseases are a major burden on human health worldwide, and little is known about how most brain diseases develop. It is believed that cardiovascular diseases can affect the function of the brain, and many brain diseases are associated with heart dysfunction, which is called the heart-brain axis. Congenital heart abnormalities with anomalous hemodynamics are common treatable cardiovascular diseases. With the development of cardiovascular surgeries and interventions, the long-term survival of patients with congenital heart abnormalities continues to improve. However, physicians have reported that patients with congenital heart abnormalities have an increased risk of brain diseases in adulthood. To understand the complex association between congenital heart abnormalities and brain diseases, the paper reviews relevant clinical literature. Studies have shown that congenital heart abnormalities are associated with most brain diseases, including stroke, migraine, dementia, infection of the central nervous system, epilepsy, white matter lesions, and affective disorders. However, whether surgeries or other interventions could benefit patients with congenital heart abnormalities and brain diseases remains unclear because of limited evidence.

## 1. Introduction

Brain diseases are a major health problem worldwide. According to the Global Burden of Disease Study Group, brain disease-related disease burden exceeds 15% of all diseases (1). At the same time, the etiology and risk factors for many brain diseases remain unclear to scientists and clinicians. Brain diseases are like a “black box”. How to subtly open the “black box” is a difficult but important problem for scientists and clinicians.

**Figure 1:**
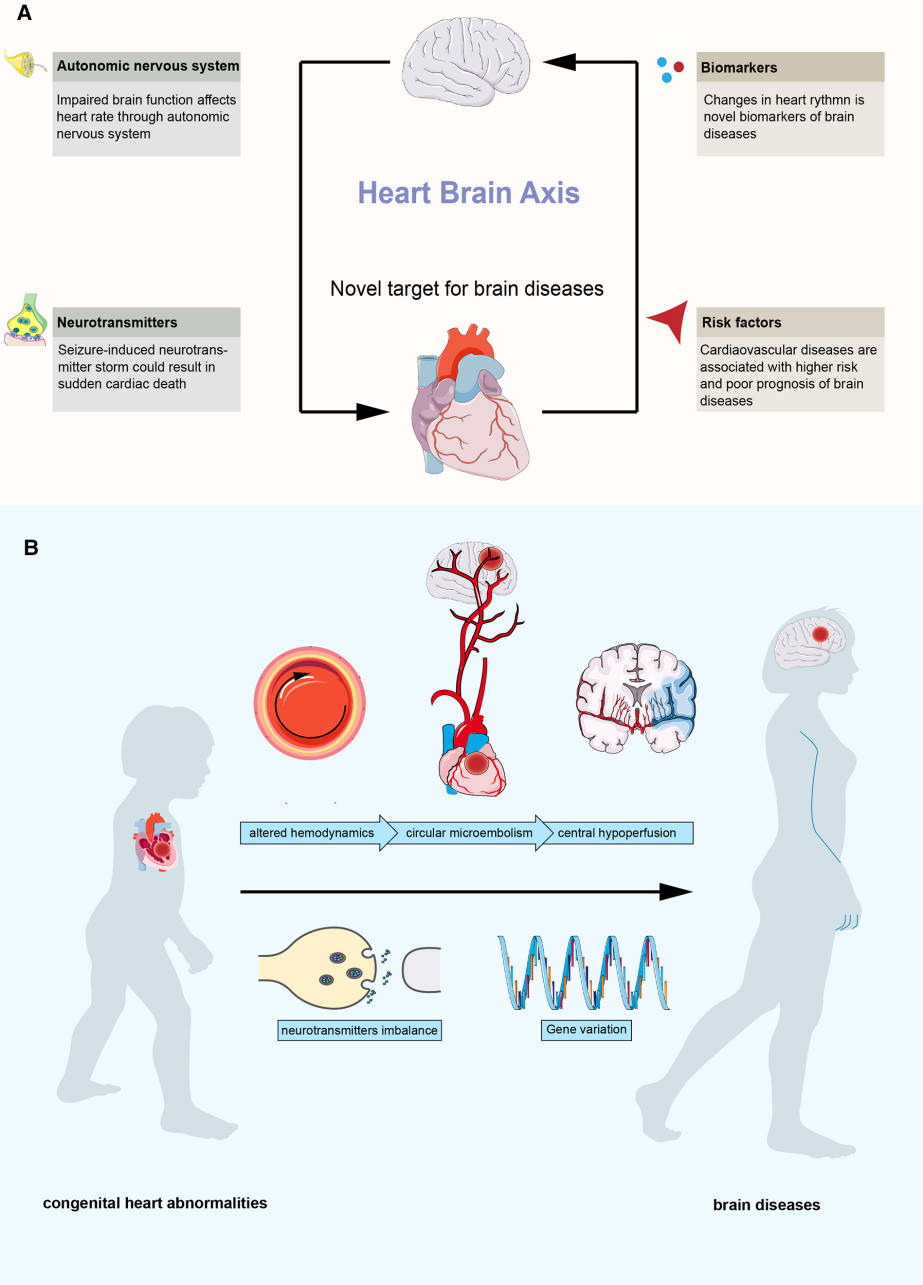
Overview of the heart-brain axis. (**A**) interactions between heart and brain. (**B**) possible mechanisms between congenital heart abnormalities and brain diseases.

The heart and the brain are the core organs of the circulatory system and the central nervous system, respectively, which play an important role in the maintenance of normal physiological functions. With the development of clinical understanding, the relationship between the heart and the brain is constantly being revealed (Figure 1A). The brain regulates the function of the heart, and impaired brain function can cause cardiovascular diseases. Studies have found that neurodegenerative diseases and affective disorders can cause the chronic failure of the autonomic nervous system and lead to arrhythmias (2). Seizures, especially generalized tonic‒clonic seizures, could result in the sustained release of neurotransmitters, including epinephrine and norepinephrine, leading to tachycardia, hypertension, and even sudden cardiac death (3). In turn, functional changes in the heart can have important effects on brain function through multiple pathways and can be risk factors or biomarkers for brain disease. Several studies have suggested that nonorganic cardiac rhythm changes can reflect abnormal brain function and be novel biomarkers for various brain diseases, including depression and posttraumatic epilepsy (4, 5). Organic heart disease, such as heart failure and atrial fibrillation, is a risk factor for many neuropsychiatric diseases, including dementia and stroke (6, 7). In addition, researchers have also found that cardiac rhythms and electroencephalogram signals are highly synchronized, further suggesting a functional connection between the heart and the brain (8). Based on the evidence, the concept of the “heart-brain axis” was proposed (9–11), and the “heart-brain axis” has become a new target for brain diseases.

Congenital heart abnormalities are an important part of cardiovascular disease, and more than 10 million newborns are born with congenital cardiovascular structural abnormalities each year (12). With the heart-brain axis, brain diseases related to congenital cardiovascular structural abnormalities have received increasing attention since the beginning of this century (13–15). In the past, it was believed that the damage to the nervous system in patients with congenital heart abnormalities was mostly attributed to surgical operations, including insufficient blood perfusion of the brain tissue during cardiopulmonary bypass, poor blood oxygen supply, and postoperative microthrombosis. With the continuous advancement of interventional therapy, open heart surgery, and cardiopulmonary bypass technology, the complications of surgery and interventional therapy continue to decrease. The long-term survival of patients with congenital heart abnormalities, especially complex congenital heart abnormalities, continues to improve. However, the situation of congenital heart abnormalities combined with an abnormal nervous system is becoming increasingly prominent, which has caused a great economic and mental burden to families and society (16–18). Current studies have found that most patients with congenital heart disease have neurological dysfunction before surgical treatment. Clinicians have found that congenital heart abnormalities, such as patent foramen ovale (PFO), could be the only abnormal finding in some patients with brain disease without any known risk factors (19–21). These cardiovascular structural abnormalities can cause abnormal intracardiac shunting, and microembolisms tend to occur at abnormal shunts due to altered hemodynamics (22, 23). The brain is the organ most susceptible to circulatory abnormalities. These microembolisms can cause central hypoperfusion, resulting in ischemic and hypoxic changes and related diseases. In addition, abnormal shunting could lead to the production-inactivation imbalance of neurotransmitters (such as serotonin), which contribute to the pathology of brain diseases (24). And several studies reported certain variations could contribute to this association (25, 26) (Figure 1B).

Many clinical studies have suggested that the onset of brain diseases related to congenital heart abnormalities mostly occurs in adulthood, which will have a serious impact on their quality of life and labor capacity (27, 28). Through evidence of the heart-brain axis, an increasing number of patients at high risk may benefit from appropriate closure of treatment (29). This article reviews common congenital heart abnormalities and their relationship with brain diseases and discusses the potential effects of their interventions on the neuropsychiatric system. Through a literature search, this paper found that congenital heart abnormalities were associated with a variety of brain diseases. In addition to stroke and migraine, central infectious diseases, white matter lesions, obstructive sleep apnea, epilepsy, and affective disorders may also be related to congenital heart abnormalities. However, the role of congenital heart abnormalities in the development of brain disease still lacks sufficient evidence. To address the question of how to provide more precise prevention and treatment for patients with congenital heart abnormalities, more high-quality evidence is needed in this field.

## 2. Congenital heart abnormalities and brain

### 2.1. Congenital heart abnormalities

Congenital heart abnormalities refer to a class of diseases that are of developmental origin and will not recover within a certain period. According to their severity, congenital heart abnormalities can be divided into simple and complex congenital heart abnormalities. Patients with complex congenital heart abnormalities often require hospitalization in a cardiovascular disease department, and the mortality and disability rates are high, such as transposition of the great arteries and tetralogy of Fallot (18, 30). Although the incidence of complex cardiovascular structural abnormalities is low, almost all complex cardiovascular heart abnormalities are fatal and often accompanied by serious complications, of which brain injury is a common extracardiac complication (31).

Common simple congenital heart abnormalities include PFO, congenital pulmonary arteriovenous fistula (PAVF), atrial septal defect (ASD), ventricular septal defect (VSD), and patent ductus arteriosus (PDA). Simple congenital heart abnormalities are less severe and generally do not affect the normal life of the patient. The incidence of simple congenital heart disease is high. It is estimated that approximately 25%–35% of adults have PFO (1). VSD, ASD, and PDA are the most common congenital heart diseases, accounting for 35.6%, 15.4%, and 10.2%, respectively (32). The incidence of PAVF has not yet been reported, but it is often underestimated because the size of lesions is too small. From the perspective of anatomical shunt, these five congenital cardiovascular structural abnormalities can be divided into right-to-left shunt and left-to-right shunt. Right-to-left shunts, including PFO and PAVF. PFO refers to a foramen ovale that has not closed after age 3 (19). PAVF refers to the congenital pulmonary arteriovenous direct connection without communication through capillaries (33). Left-to-right shunts include ASD, VSD, and PDA (34). ASD refers to the abnormality of the primordial atrial septum during embryonic development, resulting in left and right atrium openings (35). VSD refers to the development of the interventricular septum during the embryonic period, resulting in abnormal communication and left-to-right shunting at the level of the ventricle (36). PDA means that the ductus arteriosus in the fetal period continues to open after birth to form an abnormal shunt (37).

### 2.2. Crosstalk between congenital heart abnormalities and brain

Clinical evidence has demonstrated that congenital heart abnormalities are associated with pathological changes in certain brain regions. Signoriello and colleagues reported that the white matter lesion of patients with migraine and PFO was mainly in the occipital lobe compared with patients without PFO (38). And an earlier study confirmed this lobe-specific change in patients with cryptogenic stroke and PFO (39). Fontes reported that youth with congenital heart abnormalities had smaller hippocampal volumes compared with healthy controls (40). And similar pathological changes were also observed in children who underwent cardiopulmonary bypass surgery (41). Cordina and colleagues reported reduced brain volume in other brain regions, including the dorsolateral prefrontal cortex and precentral gyrus, the posterior parietal lobe and the middle temporal gyrus in adults with cyanotic congenital heart abnormalities (42). Such evidence suggests a clear relationship between congenital heart disease and pathological changes in brain regions.

Congenital heart abnormalities are associated with pathophysiological consequences, which could explain the fact that patients with congenital heart abnormalities were more likely to develop brain diseases. Hypoxia and oxidative stress are the most common mechanisms in congenital heart abnormalities-related brain diseases. Several studies reported that patients with PFO were at hypoxia state (43, 44), and hypoxia and oxidative stress were related to PFO-related many brain diseases, including stroke, migraine and epilepsy (22, 23, 45). Patients with other congenital heart abnormalities also demonstrated hypoxia and oxidative stress, and these changes could be related to abnormal neurogenesis and cortical growth (46–48). Besides, altered hemodynamics in PFO patients could be related to hypersensitivity of platelets, which could lead to abnormal 5-HT levels and prothrombotic potential (49). These mechanisms partly explain the underlying mechanisms between congenital heart abnormalities and brain diseases. However, there is still a lack of evidence to understand the complex relationship between congenital heart abnormalities and brain diseases, which needs more attention.

## 3. Right-to-left shunt and brain disease

### 3.1. PFO

PFO was the first congenital heart abnormality discovered to be related to brain diseases. It is currently believed that its pathogenesis is mainly related to paradoxical embolism caused by right-to-left shunting. Table 1 shows the clinical studies of PFO-related brain diseases, which focus on the composition ratio and odds ratio of PFO in patients with brain diseases. The relationship between PFO and stroke, especially cryptogenic stroke, is relatively clear. According to the data in the table, 47% of cryptogenic stroke patients have PFO, which is much higher than the 25%–35% in the general population. There is a clear association between PFO and migraine with aura. According to the data summarized in the table, 54% of migraine patients with aura have PFO, which is also much higher than the 25%–35% in the general population. For migraine without aura, the case‒control study by Takagi et al. showed that the prevalence of PFO was not significantly higher in patients with migraine without aura than in healthy controls (50). However, in a case‒control study by Tang et al., patients with PFO had a higher risk of migraine without aura than healthy controls (51). According to the data summarized in the table, approximately 28% of patients with migraine without aura have PFO. The relationship between PFO and migraine without aura needs more large sample studies to be explored. Evidence about PFO-related dementia is limited, but existing studies reported that dementia patients, especially Alzheimer's patients, have a higher prevalence of PFO (36.8%) (52). The prevalence of PFO was also higher in patients with obstructive sleep apnea, with data summarized in the table at 46%. However, since most studies are only cross-sectional surveys of a single group, the relationship between the two needs to be confirmed by more controlled studies.

**Table 1:** Clinical studies of PFO-related brain diseases.

Author	Year	Country	Research Type	Sample	Frequency	OR/HR
**Stroke**						
Alsheikh-Ali (27)	2009		Meta analysis	23 case‒control studies with 13,006 cases	37% (427/1,154)	OR: All ages: 2.9 (2.1 to 4.0) Young (<55yrs): 5.1 (3.3, 7.8) Old (≥55yrs): 2.0 (1.0, 3.7)
Ma (53)	2014		Meta analysis	12 case‒control studies and 6 cohort studies with 5,408 cases		OR: Case control studies: 2.94 (2.06, 4.20) Cohort studies: 1.28 (0.91, 1.80)
Giannandrea (54)	2020	UK	Cross-sectional study	1,130	22.7% (257/1,130)	
Bahl (28)	2020	UK	Cross-sectional study	167	9.6% (16/167)	
Ng (55)	2018	United States	Cohort study	182,393		OR: 2.66 (1.96, 3.63)
**Cryptogenic Stroke**						
Strambo (56)	2021	Switzerland	Cohort study	455	40% (184/455)	
Shih (57)	2021	United States	Cohort study	79	28% (7/25)	HR: 2.0 (0.4, 9.3)
Ntaios (58)	2021	Greece	Cross-sectional study	374	34% (127/374)	
West (59)	2018	United States	Cross-sectional study	712	59% (420/712)	
**Migraine**						
Takagi (50)	2016		Meta analysis	5,572	All: 44.3% (822/1,856) MA:54.7% (640/1,169) MO: 26.49% (182/687)	OR: All: 2.46 (1.55, 3.91) MA: 3.36 (1.04, 5.55) MO:1.30 (0.85, 1.99)
Zhao (60)	2021	China	Case-control study	526	All: 39.04% (98/251) MA: 48.39% (30/62) MO: 25.98% (68/189)	
Iwasaki (61)	2017	Japan	Cross-sectional study	112	All: 43.8% (49/112) MA: 54.8% (34/62) MO: 30.0% (15/50)	
Snijder (62)	2016	Netherlands	Case‒control study	887	All: 26.3% (44/168) MA: 37.5% (27/72) MO: 17.7% (17/96)	
Larrosa (63)	2016	Spain	Cross-sectional study	183	All: 53.6% (98/183)	
Tang (51)	2022	China	Case‒control study	3,741	MO:12.83%^a^ (113/881)	OR: MO:1.71 (1.19, 2.47)
**Dementia**						
Purandare (64)	2005	UK	Case-control study	57	All: 61% (25/61) AD: 62.5% (15/24) VaD: 59% (10/17)	OR: All: 2.0 (0.6, 6.5) AD: 2.1 (0.6, 7.8) VaD: 1.8 (0.5, 7.3)
Purandare (52)	2008	UK	Cross-sectional study	108	All: 33.3% (36/108) AD: 36.8% (21/57) VaD: 29.4% (15/51)	
**Obstructive sleep apnea**						
Shaikh (65)	2013	UK	Case‒control study	150	43% (43/100)	
Mojadidi (66)	2015	United States	Case‒control study	300	42% (42/100)	
Lau (67)	2013	Australia	Case‒control study	152	47.1% (48/102)	OR: 2.53 (1.20, 5.31)
Guchlerner (68)	2012	Germany	Cross-sectional study	100	72% (72/100)	
Beelke (69)	2003	Italy	Case‒control study	167	27% (21/78)	
Rimoldi (70)	2015	United States	Cross-sectional study	51	33% (17/51)	

Frequency: defined as the number of patients with PFO/the number of patients with neurological diseases.

MA: migraine with aura; MO: migraine without aura; AD: Alzheimer's disease; VaD: vascular dementia.

^a^ Defined as the number of patients with neurological diseases/the number of patients with PFO.

In addition to those listed in the table, Higuchi et al. reported a case of an intramedullary abscess spread through a PFO after dental treatment in 2011. The experience of this 51-year-old man may suggest that PFO also plays a role in central system infectious diseases (71). In addition, Italian researchers found that white matter lesions caused by PFO-related migraine are mostly located in the occipital lobe, which may suggest that white matter lesions caused by PFO have certain spatial characteristics (38). However, the sample size of this study was small (*n* = 31), and more large-sample studies are needed to confirm this correlation. PFO may also play a role in affective disorders and personality disorders. The Korean research team's study of 19-year-old youth (*n* = 511) found that patients with congenital heart abnormalities (including PFO, ASD, VSD, PDA) had higher scores in anxiety, depression, somatization symptoms, and personality disorder than the control group (72). However, this result should be treated carefully, as the study only used the score as the outcome rather than a rigorous clinical diagnosis.

### 3.2. PAVF

PAVF refers to the existence of abnormal blood vessels between the pulmonary artery and the pulmonary vein, which are directly connected to form a pulmonary vascular malformation. It is generally believed that PAVF is a relatively benign structural abnormality. Only large-sized PAVF with a diameter of more than 2 cm or a feeding artery diameter of more than 3 mm may have obvious symptoms and require active intervention (73, 74). However, as an increasing number of clinical cases of small-sized PAVF are reported, the opinion that small-sized PAVF is pathogenic has also been proposed. Table 2 summarizes case reports of brain diseases in which PAVF is a major causative factor. As seen from the table, PAVF with nodules less than 3 cm in diameter also became the only causative factor for brain diseases, including stroke, migraine, and brain abscess. In addition, two retrospective studies reported that in patients with acute ischemic stroke, excluding other risk factors, PAVF-related stroke accounted for 0.02% and 0.5%, respectively (75, 76). Moreover, the age of PAVF-related stroke patients is significantly smaller than that of other stroke patients, suggesting that PAVF-related stroke may be independent of other strokes in clinical characteristics (75). Hence, PAVF could be a novel risk factor for stroke.

**Table 2:** Case reports of PAVF-related brain diseases.

Author	Year	Country	Gender	Age	Size of PAVF	comorbidity	Description
**Stroke**							
Sousa (77)	2017	Portugal	F	46	A:1.7 mm N:3 cm × 1.4 cm V:1.9 mm	Migraine; TIA	**Symptom:** transitory right hemiplegia and language disturbance of sudden onset **Treatment:** PAVF was embolized with micro coils **Follow-up:** normal with no right-to-left shunt
Mehrbod (78)	2013	Iran	M	40	Not reported	epilepsy	**Symptom:** sudden diplopia and dysarthria accompanied by facial palsy **Treatment:** surgery closure of PAVF and anti-coagulants **Follow-up**: neurological symptoms were minimized
Fateh-Moghadam (79)	2007	Germany	M	66	Medium size	PFO Headache Dizziness	**Symptom:** recurrent stroke, after the closure of PFO, residual right-to-left shunt still present **Treatment:** closure of PAVF **Follow-up:** improvement of headache and dizziness
Felix (80)	2008	France	M	17	Not reported	HHT	**Symptom:** acute isolated aphasia. **Treatment:** PAVF was embolized with micro coils **Follow-up:** no neurologic symptoms were reported
Tomelleri (81)	2008	Italy	M	19	N: 2 cm in diameter	None	**Symptom**: vertigo, nausea, dysarthria, and left faciobrachial paresis; **Treatment:** embolization of the fistula **Follow-up:** no neurological recurrences
Peters (82)	2005	Germany	W	41	N: 4–5 mm in diameter	TIA; Stroke; PFO closure	**Symptom:** clinical symptoms of TIA **Treatment:** embolization of the fistula **Follow-up**: normal
Wozniak (83)	2015	Poland	M	15	N:7 mm in diameter	PFO	**Symptom:** two episodes of TIAs in a year, dysarthria, and paresis of the right upper and lower limb **Treatment:** Closure of both PFO and PAVF **Follow-up:** no recurrence of TIA
Sen (84)	2021	UK	W	49	A: 6–7 mm	Migraine with aura; TIAs	**Symptom:** vertical diplopia associated with right facial paraesthesia, mild slurred speech, deafness in her right ear, and weeping from the right eye; **Treatment:** closure of PAVF **Follow-up:** dramatic improvement in her migraine symptoms without any neurological event
**Migraine**							
Kakeshita (85)	2020	Japan	W	41	N:1.7 cm	stroke	**Symptom:** migraine with visual aura once or twice every month for more than 20 years; **Treatment:** Closure of PAVF; **Follow-up:** migraine completely stopped
**Brain abscess**							
Shioya (86)	2004	Japan	M	52	Not reported	Previous brain abscess	**Symptom:** sudden stroke-like onset of right hemiparesis, right hemiparesthesia, dysarthria, and sensory aphasia; **Treatment:** treated with aspiration and drainage, and the residual mass was resected; **Follow-up:** normal

F: Female; M: male; A: feeding artery; N: nidus; V: drainage vein; HHT: hereditary hemorrhagic telangiectasia; TIA: transient ischemic attack; PFO: patent foramen ovale.

In clinical practice, some PFO patients have comorbid PAVF, and PAVF in these patients is often detected due to the discovery of residual shunts after PFO closure. Both PFO and PAVF can cause right-to-left shunting, but the relationship between the two is still unclear. In a large retrospective study, Topiwala et al. found that PFO was a risk factor for PAVF-related stroke, suggesting a synergistic effect between the two (75). However, within a certain range of defects, who is dominant and how to accurately understand the synergy between the two still lacks sufficient evidence. In addition, how to simultaneously diagnose both PFO and PAVF in patients with comorbid PFO and PAVF using one auxiliary test remains a clinical challenge. The diagnosis of PFO relies primarily on contrast ultrasonography through the chest wall or the esophagus. Although ultrasound can also indicate the presence of PAVF (87), the current gold standard for the diagnosis of PAVF is contrast-enhanced chest CT (33).

## 4. Left-to-right shunt and brain diseases

### 4.1. ASD

ASD is a representative disease of left-to-right shunt congenital heart abnormalities. Table 3 summarizes current clinical studies of ASD-related brain diseases. ASD is associated with stroke and migraine. Several studies have demonstrated that patients with ASD have a significantly increased risk of stroke and migraine. Based on the data summarized in the table, 2.9% of patients with ASD had stroke [0.13% in the general population (1)], and 7.1% of patients with ASD had migraine [1.46% in the general population (1)]. For stroke, a study from Taiwan, China, showed that ASD patients were less likely to have cardiovascular events after repair, suggesting that ASD repair is beneficial for preventing cardiovascular events (88). For migraine, Magalhães et al. showed that ASD patients with migraine had a greater proportion of migraine with aura (89). However, Kato et al. reported a greater proportion of migraine without aura (90). Further research is needed to determine the relationship between ASD and migraine with and without aura.

**Table 3:** Clinical studies of ASD-related brain diseases.

Author	Year	Country	Research Type	Sample	Frequency	OR/HR
**Stroke**						
Kitamura (91)	2018	Japan	Cross-sectional study	186	1.6%^a^ (3/186)	
Karunanithi (92)	2017	Denmark	Cohort study	12,218	2.9% (14/1,111)	HR: 3.8 (2.0, 6.9)
**Migraine**						
Nyboe (93)	2019	Denmark	Cohort study	25,033	2.4% (54/2,277)	HR: 3.4 (2.5, 4.6)
Magalhães (89)	2005	Poland	Case‒control study	68	All:79.4% (27/34) MA: 65.1% (22/34) MO: 14.7% (5/34)	OR: 4.3 (1.0, 8.8)
Liu (94)	2018	China	Case‒control study	441	All: 19.1% (92/303)	
Kato (90)	2013	Japan	cross-sectional study	95	All: 24.2% (23/95) MA: 6.3% (6/95) MO: 21.1% (20/95)	

Frequency: defined as the number of patients with neurological diseases/the number of patients with ASD.

MA: migraine with aura; MO: migraine without aura.

^a^ Defined as the number of patients with ASD/the number of patients with neurological diseases.

For affective disorders, Udholm et al. reported that patients with small, unpatched ASD had higher depression and anxiety scores (*P* < 0.001), and approximately 17% of ASD patients were diagnosed with at least one psychiatric disorder, the most common being psychosomatic disorders and neurosis (95). This large cohort study suggests that ASD may potentially cause changes in brain function in patients. Future studies are needed to confirm the relationship between ASD and affective disorders. In summary, ASD is expected to become a new target for brain diseases.

### 4.2. VSD and PDA

The clinical symptoms of VSD and PDA are rather serious, and most patients are repaired after birth. Therefore, there are limited studies on the relationship between VSD and PDA and brain diseases. For VSD, Shuiab reported a 38-year-old patient with ischemic stroke in the occipital lobe who did not find any abnormality other than VSD (96). For migraine, a team from China reviewed the comorbidities of 72 VSD patients and found that 6 (8.3%) suffered from migraine (94). Dhoubhadel et al. reported a case of cryptococcal meningitis caused by VSD in 2021. The 12-year-old girl had normal immunity and no obvious abnormal exposure history. The attending doctor believed that VSD was the only risk factor for cryptococcal meningitis in this case (97). For PDA, Panagopoulos et al. reported a 7-year-old child with recurrent ischemic stroke with comorbid PDA and PFO (98). However, it is unclear which of these two congenital structural heart defects predominated in this patient. In addition, Ke et al. reported a family with a PRRX1 heterozygous mutation that resulted in familial PDA and atrial fibrillation caused by PRRX1 loss of function. The family is at a significantly increased risk of stroke (99). In conclusion, the relationship between VSD and PDA and brain diseases is still unclear, but it should be considered in clinical practice that VSD and PDA may be the etiology or risk factors for some difficult neuropsychiatric cases. Future research is needed to explore the relationship between VSD and PDA and brain disease. Not only unrepaired but also long-term follow-up outcomes of repaired VSD and PDA are also worthy of attention.

## 5. Complex congenital heart abnormalities

Patients with complex congenital heart abnormalities often have genetic abnormalities, and such genetic diseases are often accompanied by brain damage. For example, children with Down syndrome and DiGeorge syndrome often present with complex congenital heart abnormalities (such as tetralogy of Fallot (TOF/F4), complete transposition of great arteries (CTGA) and single ventricle (SV), and tricuspid atresia (TA)) comorbid brain damage, such as intellectual disability and leukomalacia. These patients are at extremely high risk for seizures (100). In addition, the severe abnormal hemodynamic state associated with complex congenital heart abnormalities can also lead to severe hypoxemia during the growth and development of children. Ischemic and hypoxic damage to the brain occurs at a very early age and mainly manifests as stroke and white matter damage. Due to the high lethality of complex congenital heart abnormalities and the lack of medical records, it is still unclear when patients with complex congenital heart abnormalities begin to develop brain injury. It is unclear whether *in utero* intervention during pregnancy could alleviate brain injury caused by complex congenital heart abnormalities.

## 6. Interventions for congenital heart abnormalities and brain diseases

Although there is a certain correlation between congenital heart abnormalities and brain diseases, high-quality randomized controlled trials are still needed to confirm whether the intervention of structural abnormalities helps relieve the disease and prevent recurrence. The only currently recognized effective intervention is PFO closure for secondary prevention of stroke. Closing the PFO after stroke could reduce the risk of recurrent stroke [RR 0.42 (95% CI 0.20, 0.91)] (101). In addition, a meta-analysis pointed out that PFO closure can help migraine relief, especially migraine with aura (102). Some studies suggest that PFO closure can relieve symptoms in OSA patients, but the sample size is small, and its effect is controversial (65, 103). For other congenital cardiovascular structural abnormalities, there is currently a lack of clinical studies to explore whether closure therapy helps relieve the disease and prevent recurrence.

Thanks to current advanced techniques and treatment measures, many patients choose closure therapy before symptoms related to congenital heart abnormalities appear. Whether early-life closure therapy has adverse effects on the neuropsychiatric system is a question that scientists and clinicians need to pay attention to. Table 4 lists closed therapies for simple congenital heart abnormalities and their associated complications. In general, the closed treatment of various congenital heart abnormalities is relatively safe, and the probability of complications is low (30). Arrhythmias such as atrial fibrillation may occur after intracardiac intervention for PFO, ASD, and VSD, but the incidence is low (104). Atrial fibrillation could increase the risk of stroke in patients, but no studies have shown that PFO and VSD closure can increase the risk of postoperative stroke. For ASD, a Danish team reported an increased risk of stroke after ASD closure [HR 5.0 (95% CI 2.3, 11.1)] (92). However, the Taiwanese research team reported that the risk of cardiovascular events was reduced after ASD closure (88), which is contrary to the findings of the Danish team. For PAVF, embolization closure or partial pneumonectomy is generally selected according to the size and location of the PAVF. These interventions are relatively safe, but care should be taken to minimize trauma and reduce the patient's time in bed. Thoracotomy for VSD and PDA is more traumatic, and patients are at increased risk of postoperative neurodevelopmental abnormalities during long-term follow-up, which may be related to intraoperative cardiopulmonary bypass and cerebral hypoperfusion (105).

**Table 4:** Interventions for simple congenital heart abnormalities and related complications.

CHAs	Closure	Complications
PFO	Catheter closure	• dislodgment of the device (106) • residual shunting • device-associated thrombus formation (107) • pericardial effusion • atrial fibrillation (108)
PAVF	Embolized with micro coils Partial lung resection	• atelectasis • infection
ASD	Catheter closure	• arrhythmias • device embolization • pericardial effusion • cardiac tissue erosion
VSD	Patch closure through sternotomy Catheter closure	• motor function and formal intelligence reduction (105) • behavioral and school performance difficulties • heart block (109) • arrhythmias (110) • residual shunt and hemolysis • valve complications
PDA	Surgical ligation Catheter occlusion	• residual shunts (111) • recurrent laryngeal nerve injury • diaphragmatic paralysis • embolization • infection • neurodevelopmental delay (112)

For the surgical treatment of complex heart abnormalities, due to the complicated operation, the large trauma to the patient, the long cardiopulmonary bypass time, and the need to maintain a long-term deep hypothermia state, the damage to the brain is relatively severe. One-third of children with complex congenital heart abnormalities without preoperative brain damage often develop brain diseases such as white matter lesions, stroke, epilepsy, and cognitive impairment after surgery. Among them, white matter lesions are the most common and more pronounced in children with a single ventricle, aortic malformation, and preoperative arterial hypoxia (113). Cardiopulmonary bypass is a form of circulation that diverts a patient's blood from the heart and lungs to the body. The normal physiological functions of the patient's heart and lungs, such as blood circulation, respiration, and oxygenation are temporarily replaced by extracorporeal circulation machines. During cardiopulmonary bypass, plaque fall, gas emboli, and surgical foreign bodies caused by intubation can become potential cerebrovascular emboli, causing cerebral ischemia-hypoxic injury (114, 115). Deep hypothermic circulatory arrest is commonly used in open heart surgery for complex congenital heart abnormalities. In the state of deep hypothermic circulatory arrest, the energy metabolism of the brain is depleted, causing brain damage through mechanisms such as calcium overload and excitatory amino acid toxicity (116–118). Studies have reported that the incidence of epilepsy after surgery for children with congenital heart abnormalities is 4% to 10%, and the incidence of epilepsy after surgery for complex congenital heart abnormalities is even higher (113). In addition, anesthetic use early in life may also be associated with impairment of brain function, including neuroapoptosis and neurocognitive deficits. However, the causality of this relationship is still debated (119).

In conclusion, surgery and interventional treatment of congenital heart abnormalities could have adverse effects on the brain. For simple congenital heart abnormalities, how to assess the risks brought by surgery and the risks brought by congenital heart abnormalities and properly compare the priorities of the two are the next issues to be solved. For complex heart abnormalities, surgical treatment is unavoidable. How to minimize brain damage by improving surgical procedures, preoperative intervention, and postoperative treatment is also an urgent problem to be solved (120, 121).

## 7. Discussion

This article summarizes the relationship between brain diseases and common congenital heart abnormalities. Overall, the brain disorders most associated with common congenital heart abnormalities are stroke and migraine, obstructive sleep apnea, dementia, cognitive impairment, white matter lesion, epilepsy, affective disorders, and central system infectious diseases, which may also have a certain relationship with congenital heart abnormalities. Some interventions for congenital heart abnormalities could increase the risk of brain disease. There is insufficient high-quality evidence about whether interventions should be conducted to reduce the risk of brain diseases caused by congenital heart abnormalities.

Apart from the direct relationship of the heart-brain axis, several risk factors could have an influence on both the heart and the brain. Agrimi et.al. reported that mice co-exposed to obesity and psychosocial stress displayed both cardiac and hippocampal dysfunction, which was associated with local brain-derived neurotrophic factor depletion (122). And smoking is a risk factor of both cardiovascular diseases and many brain diseases. Such evidence indicated that certain risk factors could affect the heart-brain axis (123, 124). However, whether such risk factors exist in congenital heart disease-related brain diseases remains unknown. In addition to traditional risk factors, sex or gender is believed to have an impact on the heart-brain axis, especially for the female heart-brain axis (125). For example, females tend to have poorer cognitive outcomes after cardiac operations (126). And for congenital heart diseases, PFO is believed to be a risk factor for stroke in pregnant women (127). However, an earlier study reported poorer neurodevelopmental outcomes in boys born with congenital heart disease requiring early surgical repair (128). How sex or gender influence congenital heart disease-related brain diseases requires more attention.

The heart-brain relationship has been revealed by a growing body of research (129–131). Although the brain, as a relatively independent organ, is isolated from the periphery by the blood‒brain barrier. However, it must take in necessary nutrients through the circulation, and the heart, as the central structure of the circulation, has an important influence on the circulation. Although there are only minor structural changes in congenital heart abnormalities, the impact on circulation is huge. The altered hemodynamics of abnormal shunts have corresponding effects on the brain and may ultimately result in brain disease, which is the heart-brain axis. The heart-brain axis affects the center through peripheral changes, resulting in diseases. The heart-brain axis has the potential to be a bridge to better understand brain function and diseases, enabling scientists and clinicians to unlock the “black box” of brain diseases (11, 132, 133).

The mechanism by which abnormal cardiac structure affects the function of the central nervous system still needs further study. Paradoxical embolism is currently the most mainstream mechanism theory. Abnormal hemodynamics caused by abnormal intracardiac shunts promote embolus formation (22). The emboli enter the cerebral blood vessels through circulation to form embolic ischemia and hypoxia. A hypoperfusion state can induce blood‒brain barrier damage, neuroinflammation, and abnormal neuroelectrophysiological activities, resulting in diseases (134, 135). In addition to the ischemic and hypoxic changes caused by paradoxical embolism, some scholars have suggested that the abnormal production and inactivation of serotonin may also be one of the mechanisms (24). The majority of serotonin inactivation is performed in the lungs. An abnormal intracardiac shunt changes the pulmonary circulation blood volume, which in turn leads to increased or decreased circulating serotonin. As an important neurotransmitter, the imbalance of serotonin will lead to abnormal nerve activity and cause disease (136–139). The mechanism of brain diseases related to congenital heart abnormalities is still inconclusive, and more clinical and basic research is needed to explore the above hypothesis.

Brain disease is a major threat to human health. The heart-brain axis could provide a new driving force for its early prevention and precise treatment, benefiting patients and their families worldwide. The improvement of the heart-brain axis requires the participation of more research teams. Further exploration of the important role of congenital heart abnormalities in the occurrence and development of brain diseases and the development of new therapeutic targets based on this feature can benefit more patients.
